# Widespread and tissue-specific expression of endogenous retroelements in human somatic tissues

**DOI:** 10.1186/s13073-020-00740-7

**Published:** 2020-04-28

**Authors:** Jean-David Larouche, Assya Trofimov, Leslie Hesnard, Gregory Ehx, Qingchuan Zhao, Krystel Vincent, Chantal Durette, Patrick Gendron, Jean-Philippe Laverdure, Éric Bonneil, Caroline Côté, Sébastien Lemieux, Pierre Thibault, Claude Perreault

**Affiliations:** 1 Institute of Research in Immunology and Cancer, Université de Montréal, P.O. Box 6128, Downtown Station, Montréal, QC H3C 3J7 Canada; 2 Department of Medicine, Université de Montréal, Montréal, QC Canada; 3 Department of Computer Science and Operations Research, Université de Montréal, Montréal, QC Canada; 4 Department of Biochemistry and Molecular Medicine, Université de Montréal, Montréal, QC Canada; 5 Department of Chemistry, Université de Montréal, Montréal, QC Canada; 6 Division of Hematology-Oncology, Hôpital Maisonneuve-Rosemont, Montréal, QC Canada

**Keywords:** Endogenous retroelements, Immunopeptidome, Major histocompatibility complex, Medullary thymic epithelial cells, Somatic tissues, Systems biology, Transcriptome

## Abstract

**Background:**

Endogenous retroelements (EREs) constitute about 42% of the human genome and have been implicated in common human diseases such as autoimmunity and cancer. The dominant paradigm holds that EREs are expressed in embryonic stem cells (ESCs) and germline cells but are repressed in differentiated somatic cells. Despite evidence that some EREs can be expressed at the RNA and protein levels in specific contexts, a system-level evaluation of their expression in human tissues is lacking.

**Methods:**

Using RNA sequencing data, we analyzed ERE expression in 32 human tissues and cell types, including medullary thymic epithelial cells (mTECs). A tissue specificity index was computed to identify tissue-restricted ERE families. We also analyzed the transcriptome of mTECs in wild-type and autoimmune regulator (AIRE)-deficient mice. Finally, we developed a proteogenomic workflow combining RNA sequencing and mass spectrometry (MS) in order to evaluate whether EREs might be translated and generate MHC I-associated peptides (MAP) in B-lymphoblastoid cell lines (B-LCL) from 16 individuals.

**Results:**

We report that all human tissues express EREs, but the breadth and magnitude of ERE expression are very heterogeneous from one tissue to another. ERE expression was particularly high in two MHC I-deficient tissues (ESCs and testis) and one MHC I-expressing tissue, mTECs. In mutant mice, we report that the exceptional expression of EREs in mTECs was AIRE-independent. MS analyses identified 103 non-redundant ERE-derived MAPs (ereMAPs) in B-LCLs. These ereMAPs preferentially derived from sense translation of intronic EREs. Notably, detailed analyses of their amino acid composition revealed that ERE-derived MAPs presented homology to viral MAPs.

**Conclusions:**

This study shows that ERE expression in somatic tissues is more pervasive and heterogeneous than anticipated. The high and diversified expression of EREs in mTECs and their ability to generate MAPs suggest that EREs may play an important role in the establishment of self-tolerance. The viral-like properties of ERE-derived MAPs suggest that those not expressed in mTECs can be highly immunogenic.

## Background

Endogenous retroelements (EREs) are remnants of transposable elements that successfully integrated our germline DNA millions of years ago [1, 2]. After initial integration in the genome, EREs further increased their copy number via several successive waves of retrotransposition [3, 4]. Now, most ERE sequences contain mutated or truncated open reading frames and have lost their capacity to transpose in the genome [2]. Phylogenic analyses have allowed the classification of EREs in families based on sequence homology [5, 6]. Most EREs are categorized into three groups, which altogether comprise ~ 42% of the human genome: the long terminal repeats (LTR) as well as the long and short interspersed nuclear elements (LINE and SINE) [7–9].

Hosts repress ERE expression in order to protect their genomic integrity from deleterious insertions of EREs in open reading frames [10, 11]. Indeed, a strict epigenetic regulation of ERE sequences is applied at both the DNA and histone levels [12]. Growing evidence suggests that KRAB zinc finger proteins (KZFPs) are involved in an evolutionary arms race to repress the expression of novel ERE integrations [13]. KZFPs recruit numerous restriction factors to silence ERE sequences: the histone methyltransferase SETDB1, the DNA methyltransferase proteins, the nucleosome remodeling and deacetylase complex NuRD, and the heterochromatin protein HP1 [14]. KZFP-independent mechanisms, such as the HUSH complex [15] and the histone demethylase LSD1 [16], also apply non-redundant epigenetic silencing on ERE sequences. Nevertheless, some “domesticated” EREs contribute at many levels to human development and survival. Specifically, ERE sequences are key components of several promoters and enhancers of genes implicated in interferon responses, DNA damage response in the male germline, and maintenance of stem cell pluripotency [17–19]. Additionally, a LINE-derived transcript is essential to embryonic stem cell (ESCs) self-renewal via activation of rRNA synthesis [20]. Finally, syncytins are ERE-derived proteins that mediate cell-cell fusion to allow the formation of the placental syncytium [21, 22].

The dominant paradigm holds that EREs are expressed in ESCs as well as in germline cells, but are repressed in other differentiated cells outside specific contexts in which they have relevant functions [12]. However, studies on ERE expression have been limited to subsets of ERE families in one or few tissues. Additionally, to our knowledge, no study has addressed ERE expression in the thymus where central T cell immune tolerance is established. Hence, we have no clue as to the ability of EREs to induce T cell tolerance. In the present report, we demonstrate that ERE expression is widespread in human tissues, but with tissue-specific profiles. In addition, our mass spectrometry (MS) analyses revealed that the three main groups of EREs generate MHC I-associated peptides (MAPs) retaining similarities with viral peptides. Finally, we found that mTECs express top levels of EREs, in a fashion that is independent of the autoimmune regulator (AIRE), which could mediate self-tolerance to the antigens deriving from them.

## Methods

### Transcriptomic data manifest

RNA-seq data of 30 non-redundant human tissues were downloaded from the Genotype-Tissue Expression (GTEx) on the dbGaP portal (accession number phs000424.v8.p2.c1) [23]. When possible, 50 samples were randomly selected per tissue; otherwise, all available samples were analyzed. Transcriptomic data of ESCs from Lister et al. [24] were downloaded from the sequence read archive. RNA-seq data of purified hematopoietic cells were obtained from the Gene Expression Omnibus (GEO) (projects PRJNA384650 and PRJNA225999) [25, 26]. Six human mTEC samples were analyzed: four from Laumont et al. [27] and two additional samples processed with the same protocol with minor modifications: (i) after transfer to our laboratory, thymic samples were frozen in cryovials containing a cryoprotective medium composed of 5% DMSO and 95% Dextran-40 solution (5% concentration); (ii) CD45^−^ cells were magnetically enriched with the CD45 Microbeads human kit from Miltenyi Biotec (no. 130-045-801) prior to mTEC sorting; (iii) cDNA libraries were prepared with the KAPA mRNAseq stranded kit (KAPA, Cat no. KK8421); and (iv) sequencing generated around 400 × 10^6^ reads per sample. Transcriptomic data of the two new mTEC samples were deposited on the Gene Expression Omnibus (GEO) as GSE127826 [28]. For the complete list of human samples analyzed, see Table S1 (Additional file 1: Table S1). Mature murine mTECs (mTEC^hi^) data were obtained from St-Pierre et al. [29] on GEO (accession GSE65617).

### Expression of transcripts derived from EREs and canonical genes

RNA-seq reads of human samples were trimmed with Trimmomatic *0.35* [30] to remove adapters and low-quality sequences. Expression levels of transcripts and EREs were quantified in transcripts per million (TPM) with kallisto *0.43.1* [31] with indexes composed of (i) Ensembl 88 (GRCh38.88) transcripts and human ERE sequences from RepeatMasker or (ii) Mouse mm10 (GRCm38) transcripts and murine ERE sequences from RepeatMasker for human and murine samples, respectively. TPM values of transcripts and ERE sequences were summed in genes and ERE families based on Ensembl and RepeatMasker annotations, respectively, using the aggregate function in R.

### ERE expression profiling in human tissues

Expression levels of ERE families were computed for each tissue by calculating the median expression across all samples for a given tissue. The numbers of standard deviations from the mean (row *Z*-score) of ERE families for each tissue were determined using the scale function in R. The Euclidean distance was then calculated between all tissues based on the row *Z*-scores of ERE families, followed by an unsupervised hierarchical clustering. The pvClust package in R [32] was used to assess the statistical significance of the clustering using a bootstrap procedure (1000 iterations). Finally, standard deviations of expression of each ERE family between samples of a given tissue were computed.

### Quintile ranking of ERE expression in somatic tissues

Median expression of ERE families were calculated among all samples of a given tissue. Tissues were then ranked based on their expression level of each ERE family individually and assigned to quintiles of 6, 6, 8, 6, and 6 tissues. Finally, tissues were sorted based on the number of times they were assigned to the fifth quintile.

### Identification and characterization of tissue-restricted EREs (TREs)

The τ index of tissue specificity was calculated as per Yanai et al. [33]. Briefly, the τ index is defined as:

$$ \tau =\frac{\varSigma_{i=1}^N\left(1-{x}_i\right)}{N-1} $$

where *x*_*i*_ is the level of expression of a gene or ERE family in tissue *i* normalized to its maximal expression level among all tissues, and *N* is the number of tissues. Genes and ERE families with τ ≥ 0.8 were considered as tissue-restricted. To determine in which tissue(s) a tissue-restricted gene or ERE family was overexpressed, a binary pattern was computed as reported by Yanai et al. [33]. Briefly, tissues were sorted based on their expression level for each tissue-restricted gene (TRG) or ERE family (TRE). The distance between neighboring tissues was calculated, and the maximal distance or “gap” was used as a threshold for the binary pattern. Tissues with an expression level above the gap were considered as overexpressing the TRG or TRE while other tissues were considered as underexpressing them, and were given a value of 1 or 0, respectively. ERE groups were determined for all identified TREs, and the proportions of LINE, LTR, and SINE elements in TREs were compared to their representation among ERE families. A chi-squared test was performed to assess the enrichment of discrete ERE groups among TREs. Using the above-described binary pattern, the number of overexpressing tissues was determined for each TRG or TRE.

### Impact of AIRE on ERE expression in mTECs

Lists of AIRE-dependent, AIRE-independent, and constitutively expressed genes were generated as per St-Pierre et al. [29]. Expression levels of these three sets of genes as well as ERE families were compared between wild-type (*n* = 3) and AIRE knock-out (*n* = 3) murine mTEC^hi^ using Wilcoxon tests. Expression levels of each individual ERE family were also compared between wild-type and AIRE knock-out mice using Wilcoxon tests.

### MS analyses

Immunopeptidomic data of a cohort of 16 B-lymphoblastoid cell line (B-LCL) samples from Pearson et al. [34] were downloaded from the PRIDE Archive (Project PXD004023). For the detailed protocol of mild acid elution and peptide processing, see Granados et al. [35]. Peptides were identified using Peaks X (Bioinformatics Solution Inc.), and peptide sequences were searched against the personalized proteome of each sample. For peptide identification, tolerance was set at 5 ppm and 0.02 Da for precursor and fragment ions, respectively. The occurrence of oxidation (M) and deamination (NQ) was considered as post-translational modifications.

### Identification of ereMAPs

For individual B-LCL samples, RNA-seq reads were aligned to the Ensembl 88 human reference genome (GRCh38.88) using STAR [36] with default parameters. Using the intersect mode of the BEDTools suite [37], reads entirely mapping in RepeatMasker and Ensembl annotations were separated in ERE and canonical datasets, respectively, and any read seen in the canonical dataset was discarded from the ERE dataset*.* Unmapped reads, secondary alignments, and low-quality reads were then removed from the ERE dataset using Samtools view [38] with the following parameters: -f “163”, “147”, “99” or “83”, and -F “3852”. In order to keep a manageable database size, ambiguous nucleotides were trimmed from reads of the ERE dataset, followed by a translation in all possible reading frames. Finally, the resulting ERE amino acid sequences were spliced to remove sequences following stop codons. Only sequences of at least 8 amino acids were kept and given a unique ID to generate a theoretical ERE proteome. In parallel, a canonical personalized proteome containing the polymorphisms of the donor was generated as per [27] for each sample. Briefly, single-nucleotide variants were detected using freebayes version 1.0.2 [39], and variants with a minimal alternate count of 5 were inserted in transcript sequences using pyGeno [40]. Expression levels of transcripts were quantified with kallisto using GRCh38.88 transcripts (downloaded from Ensembl) as an index, and only transcripts with a TPM > 0 were translated into a canonical proteome, which was concatenated with the ERE proteome to generate a personalized proteome unique to each sample. To further validate our proteogenomic workflow, we also analyzed matched transcriptomic and immunopeptidomic data of an ovarian cancer cell line (OVCAR-3) treated with IFNγ (12.5 ng/mL) for 72 h in order to increase MHC I expression. Transcriptomic and immunopeptidomic data of OVCAR-3 cells were deposited on GEO as GSE147570 (BioProject accession number: PRJNA615537) [41] and on the PRIDE Archive (Project PXD018124) [42], respectively.

### Peptide annotation and validation

Following peptide identification, a list of unique peptides was extracted for each sample, and a false discovery rate (FDR) of 5% was applied to the peptide scores. Binding affinities to the sample’s HLA alleles were predicted with NetMHC4.0 [43] or with NetMHCpan-4.0 [44] when an HLA allele was not included in NetMHC4.0, and only 8- to 11-amino-acid-long peptides with a percentile rank ≤ 2% were included for further annotation. For each peptide, a binary code was generated based on the presence or absence of its amino acid sequence in the ERE and canonical proteomes, and an ERE status of “yes,” “maybe,” or “no” was given to the peptide accordingly. Peptides that were seen only in the ERE proteome or the canonical proteome were classified as “yes” and “no,” respectively. To determine if candidates with a “maybe” status were ereMAP candidates, we retrieved all their possible nucleotide coding sequences from the sample’s reads and split them in a set of 24-nucleotide-long subsequences (*k*-mers). These *k*-mers were then queried in 24-nucleotide-long *k*-mer databases generated from our ERE and canonical reads datasets using Jellyfish version 2.2.3 [45] (with the -C argument to consider the read’s sequence and its reverse complement). Only peptides encoded by more than one read were kept for further validation to reduce risks of sequencing errors. If at least one of the MAP-coding sequences (MCS) was only seen in the canonical read dataset, the peptide was discarded. “Maybe” peptides were considered as ereMAP candidates if the minimal occurrence of their most abundant MCS was at least 10 times higher in the ERE *k*-mer database than in the canonical *k*-mer database. Because leucine and isoleucine variants are not distinguishable by standard MS approaches, all possible I/L variants for each ereMAP candidates were searched in the personalized proteome. If one of the I/L variants had a higher expression in the personalized proteome, the ereMAP candidate was discarded. The genomic region generating each ereMAP candidate was determined by mapping the reads coding for the peptide on the GRCh38.88 assembly of the reference genome with the BLAT algorithm of the UCSC Genome Browser. If a clear genomic region could not be found, the peptide was discarded. Genomic regions coding for ereMAP candidates were then inspected in IGV [46] to see if the MCS contained known germline polymorphisms (using dbSNP v.149), and candidates were kept or discarded based on their orientation in ERE and annotated sequences. Briefly, any ereMAP candidate whose MCS mapped in the sense of a gene coding sequence was discarded, whereas candidates whose coding sequences mapped in intergenic regions were considered as ereMAPs no matter their orientation. Candidates were also discarded if they fulfilled these two conditions: (i) their MCS mapped in the sense of an intron and in the antisense of the ERE and (ii) if their MCS did not map in other ERE sequences (Additional file 2: Fig. S3). Finally, the MS/MS spectra of the ereMAP candidates were manually validated to ensure the quality of the identification. Peptides that passed all these validation steps were then considered as ereMAPs.

### Characterization of ereMAPs

During manual validation in IGV, characteristics regarding the family and group of the ERE generating the peptides, the type of genomic region encoding the peptide (coding sequence, intronic, or intergenic), and the orientation of the peptide sequences (sense or antisense) were retrieved for individual ereMAPs. When a peptide was identified in multiple samples and had different characteristics depending upon the sample, all possibilities were kept; otherwise, they were aggregated to reduce redundancy. The expression levels of ERE families that were source or non-source of ereMAPs were averaged among B-LCL samples, and their distributions were compared with a Mann-Whitney test. We next compared the proportions of the three main groups of EREs (LINE, LTR, and SINE) in the genome, transcriptome, and immunopeptidome. Representation of EREs in the transcriptome was assessed in our B-LCL samples: the expression levels of LINE, LTR, and SINE elements were summed in each sample and divided by the expression level of all EREs. We then averaged these transcriptomic proportions across all B-LCL samples. We used immunopeptidomic proportions of LINE, LTR, and SINE elements from the ereMAPs identified in this work, whereas the genomic proportions were taken from Treangen et al. [8]. A chi-squared test was performed to compare the proportions of ERE groups at the genomic, transcriptomic, and immunopeptidomic levels. The proportions of ERE sequences located in intergenic and intronic regions as well as in coding sequences were determined by intersecting the genomic localization of ERE sequences with the localization of introns and exons from the UCSC Table Browser (files downloaded on August 21, 2019). A chi-squared test was used to determine the enrichment of a certain genomic region for ereMAP generation. Last, Kendall tau correlation between the number of ereMAPs generated by each ERE family and the number of copies of the family’s sequence in the human genome (determined from RepeatMasker annotations) was computed with a confidence level of 95%.

### Expression profiling of ereMAPs’ coding sequences

To evaluate the expression of the ereMAP-coding sequences in peripheral tissues, we downloaded RNA-seq data of 30 tissues from the GTEx Consortium (phs000424.v7.p2). For the complete protocol of this analysis, see Laumont et al. [27]. Briefly, we generated 24-nucleotide-long *k*-mer databases for each sample, in which we queried each ereMAP-coding sequence’s 24-nucleotide-long *k*-mer set. For each ereMAP, the minimal occurrence in the *k*-mer set was used as the number of reads coding for the peptide in a given sample (*r*_overlap_). The number of reads coding for a peptide was normalized between RNA-seq experiments by dividing *r*_overlap_ by the total number of reads of the sample and multiplying this number by 10^8^ to obtain the number of reads detected per hundred million reads sequenced (rphm). We then averaged the log-transformed rphm values (log_10_(rphm + 1)) for each tissue, and an average expression superior to 10 rphm in a tissue was considered as significant. This analysis was also performed on 12 TCGA cohorts (50 randomly selected samples per cohort) to assess the expression of transcripts coding our ereMAPs (identified in B-LCLs) in the following cancer types: urothelial bladder carcinoma, breast invasive carcinoma, colon adenocarcinoma, head-neck squamous cell carcinoma, kidney renal clear cell carcinoma, liver hepatocellular carcinoma, lung adenocarcinoma, lung squamous cell carcinoma, ovarian cancer, pancreatic adenocarcinoma, prostate adenocarcinoma, and skin cutaneous melanoma. Last, methylation data (HM27 array for ovarian cancer, HM450 for other cancer types) matched with the RNA-seq samples used to profile ereMAPs’ expression in TCGA cohorts were downloaded when available. Only probes located in a window of 5000 nucleotides from the ereMAPs’ genomic locations were used for this analysis. We then computed the Pearson correlation between the ereMAP’s RNA expression (in rphm) and the methylation level of the genomic region coding for the peptide.

### Amino acid composition of ereMAPs

In addition to the list of ereMAPs identified on our B-LCL samples, two linear and MHC I-restricted epitopes’ sequence datasets were downloaded from the Immune Epitope Database: the first dataset consists of 36,472 MAPs from any virus infecting human cells, and the second one consists of 282,069 human canonical MAPs (downloaded on August 7, 2019). Lists of 8- to 11-amino-acid-long MAPs were extracted from these two datasets. The usage frequency of each amino acid was calculated by dividing their occurrences by the total number of amino acids in the ERE, viral, and human canonical MAP datasets. In parallel, datasets were separated in subsets of 8-, 9-, 10-, and 11-amino-acid-long MAPs, and frequencies of amino acids were computed for each peptide position of each subset of MAPs. The 11-amino-acid-long MAP subset was discarded because of an insufficient number of ereMAPs (*n* = 2).

### Viral homology

To assess the similarity between ereMAPs and viral peptides, we used the same datasets of viral and human canonical MAPs from the Immune Epitope Database used for the amino acid composition analysis (see the “Amino acid composition of ereMAPs” section). We aligned ereMAP sequences to this database of viral peptides using version 2.2.28 of the Protein Basic Local Alignment Tool (BLASTp) [47] in the blastp-short mode with the following arguments: -word_size 2, -gapopen 5, -gapextend 2, -matrix PAM30, and -evalue 10 000 000. As control, human canonical MAPs were aligned to the viral peptide dataset with BLASTp. For the viral homology analysis, we compared the 103 ERE MAPs to 10,000 groups of 103 randomly sampled canonical MAPs. We calculated the percentage of identity (%_*I*_) of ereMAPs and canonical MAPs with viral peptides as:

$$ {\%}_I=\frac{M_{\mathrm{max}}\times {L}_{\mathrm{a}}}{L_{\mathrm{p}}}\times 100\% $$

where *M*_max_ is the maximal percentage of identical matches with the viral MAP database, *L*_a_ is the length of the alignment, and *L*_p_ is the length of the ereMAP or the canonical MAP. The average percentage of identity of ereMAPs and each subgroup of the bootstrap distribution was computed, and the *P* value was determined as the number of times that the percentage of identity of the bootstrap distribution was higher than the percentage of identity of ereMAPs divided by the number of bootstrap iterations (10,000) as per Granados et al. [48].

### ereMAPs’ immunogenicity prediction

We used the Repitope algorithm [49] with default settings to predict ereMAPs’ immunogenicity for CD8 T cells. As negative controls, we used conventional thymic MAPs identified by Adamopoulou et al. [50]. The distributions of immunogenicity scores for thymic MAPs and ereMAPs were compared with a Mann-Whitney test.

### Generation of monocyte-derived dendritic cells (DCs)

Monocyte-derived DCs were generated from frozen PBMCs, as previously described [51, 52]. Briefly, DCs were prepared from the adherent PBMC fraction by culture for 8 days in X-vivo 15 medium (Lonza Bioscience) complemented with 5% human serum (Sigma-Aldrich), sodium pyruvate (1 mM), IL-4 (100 ng/mL, Peprotech), and GM-CSF (100 ng/mL, Peprotech). After 7 days of culture, DCs were matured overnight with IFNγ (1000 IU/mL, Gibco) and LPS (100 ng/mL, Sigma Aldrich). DCs were loaded with 2 μg/mL of peptide during 2 h after maturation process and were then irradiated (40 Gy) before they were used as APCs in T-DC culture. As control, the experiment was performed for the so-called MelanA peptide when the number of T cells was sufficient. This peptide (A**L**PVALPSL) is an in vitro modified version of the wild-type E***A***AGIGILTV MART-1/Melan-A26-35 decamer and is one of the most immunogenic human MAPs.

### In vitro peptide-specific T cell expansion

Peptide-specific CD8+ T cells were expanded as previously described, with some minor modifications [52, 53]. Briefly, thawed PBMCs were first CD8^+^ T cell enriched using the Human CD8^+^ T cell isolation kit (Miltenyi Biotech) and co-incubated with autologous peptide-pulsed DCs at a DC:T cell ratio of 1:10. Expanding T cells were cultured for 4 weeks (with pulsed-DC stimulation every 7 days) in Advanced RPMI medium (Gibco) supplemented with 8% human serum (Sigma-Aldrich), l-glutamine (Gibco), and cytokines. For the first coculture week, IL-12 (10 ng/mL) and IL-21 (30 ng/mL) were added to the medium. Two days after, IL-2 (100 UI/mL) was also added to the cytokine mix. In the second week, IL-2 (100 UI/mL), IL-7 (10 ng/mL), IL-15 (5 ng/mL), and IL-21 (30 ng/ml) were added to the medium. For the two last weeks of coculture, IL-2 (100 UI/mL), IL-7 (10 ng/mL), and IL-15 (5 ng/mL) were used. Medium supplemented with the appropriate cytokine mix was added in the cocultures every 2 days. At the end of the fourth week of coculture, cells were harvested in order to perform ELISPOT assays. If the number of specific T cells was not sufficient at the end of the fourth week of coculture, cocultures were maintained for an additional week (week 5).

### IFNγ ELISPOT assay

ELISpot Human IFNγ (R&D Systems, USA) kit was used according to the manufacturer’s recommendations. Harvested CD8+ T cells were then plated and incubated at 37 °C for 24 h in the presence of irradiated peptide-pulsed PBMCs (40 Gy) that were used as stimulator cells. As a negative control, sorted CD8 T cells were incubated with irradiated non-pulsed PBMCs. Spots were revealed as mentioned in the manufacturer’s protocol and were counted using an ImmunoSpot S5 UV Analyzer (Cellular Technology Ltd., Shaker Heights, OH). IFNγ production was expressed as the number of peptide-specific spot-forming cells (SFC) per 10^6^ CD8+ T cells after subtracting the spot counts from negative control wells.

## Results

### Expression of ERE transcripts in normal human tissues and cells

**Fig. 1:**
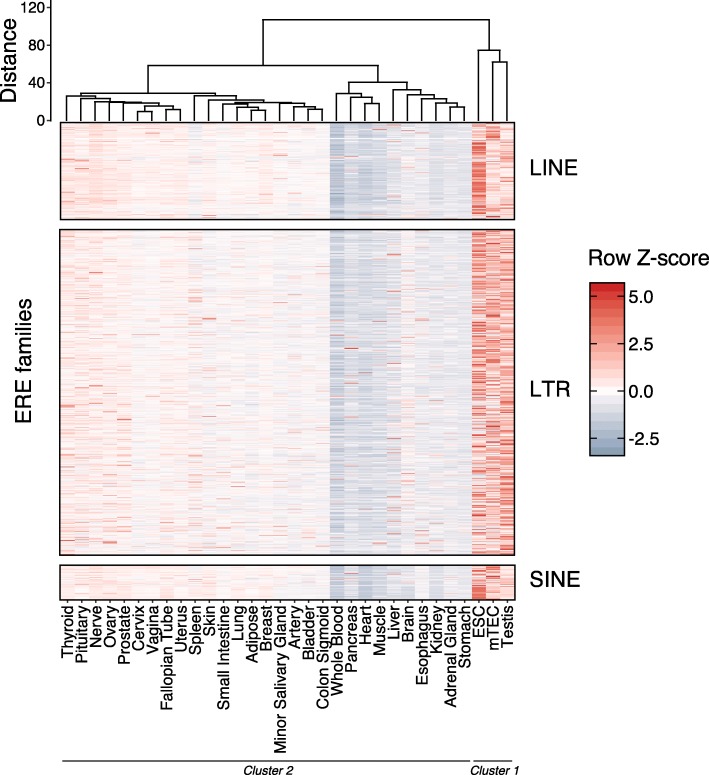
Expression profiling of endogenous retroelements in 30 healthy human tissues and 2 cell types. Hierarchical clustering of tissues based on the expression levels of the 809 ERE families sorted in LINE, LTR, and SINE elements. For each tissue, the mean expression of ERE families was computed among available samples. Row *Z*-scores were then determined for each ERE family across tissues. Significant clusters identified by pvClust are indicated (cluster 1 and cluster 2)

To assess ERE expression in healthy human tissues, we quantified the expression levels of the 809 ERE families contained in the RepeatMasker annotations in 1371 samples from 30 different healthy human tissues and 2 cell types (mTECs and ESCs). For brevity, mTECs and ESCs will be referred to as tissues in the rest of the manuscript. We calculated the median expression of each ERE family among samples of a given tissue (Additional file 1: Table S2) and then computed the row *Z*-score across tissues. Unsupervised hierarchical clustering identified a statistically significant cluster of three cell types with high ERE expression: ESCs, testis, and mTECs (Fig. 1). The remaining tissues could then be visually separated into two groups with low and intermediate ERE expression (Fig. 1). High ERE expression (cluster 1) in ESCs and testis was expected. The salient finding was the high ERE expression in mTECs which, to the best of our knowledge, has never been reported before. Comparison with hematopoietic cell types at several differentiation stages confirmed the high ERE expression in mTECs and ESCs (Additional file 2: Fig. S1A). Computing the standard deviation of ERE expression among individual samples for each tissue also revealed that most ERE families displayed low interindividual variability (Additional file 2: Fig. S1B). Finally, while quintile ranking analysis showed that ERE expression was generally concordant between ERE families in each tissue analyzed, almost all tissues expressed some ERE families at high levels (Additional file 2: Fig. S2), suggesting that some tissue-specific factors regulate ERE expression in human tissues.

### Most human tissues show a tissue-specific ERE expression

**Fig. 2:**
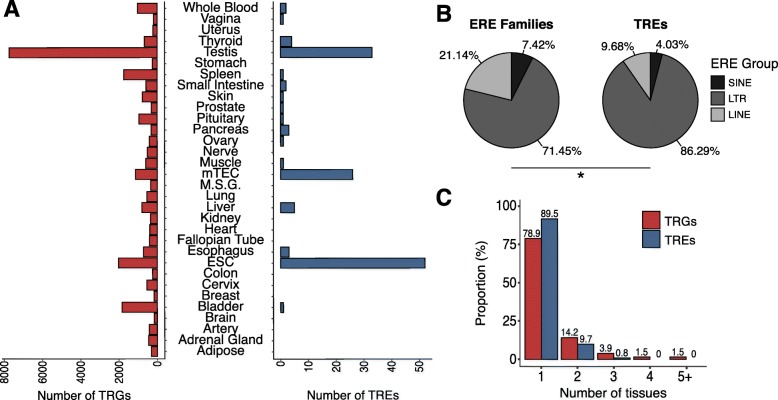
Tissue specificity of ERE expression in healthy human tissues. Tissue specificity indexes were computed for ERE families as well as annotated genes. **a** Barplots showing the number of TRGs and TREs for each of the 32 healthy human tissues analyzed. **b** Pie charts depicting the proportions of the 809 ERE families (left panel) or TREs (right panel) belonging to the LINE, LTR, and SINE groups (chi-squared test, *P ≤ 0.05). **c** Histogram showing the number of tissues in which each identified TRGs and TREs are overexpressed

To ascertain if the expression of discrete ERE families was restricted to specific tissues, we computed the τ index of tissue specificity as defined by Yanai et al. [33]. Briefly, the τ index compares the expression of a gene in a set of tissues and has a value ≤ 0.4 for housekeeping genes and ≥ 0.8 for tissue-restricted genes [54]. We identified a total of 124 ERE families with a tissue-restricted expression. As a control, we computed the τ index for annotated genes and known tissue-restricted genes (TRGs), such as *INS*, *CRP*, and *CHRNA1*. The majority (108/124) of the tissue-restricted ERE families (TREs) were identified in ESCs, testis, and mTECs, revealing that in addition to their high expression of EREs, these tissues express a broader repertoire of EREs than other tissues (Fig. 1, Fig. 2a). Nonetheless, tissue-restricted expression of EREs is a widespread phenomenon across human tissues because we identified TREs in 17 out of the 32 human tissues analyzed. For a given tissue, the number of TREs is positively associated with the number of TRGs (Fig. 2a) suggesting some commonality between expression regulation of TRGs and TREs. We also identified a significant enrichment of LTRs in TREs (86.29%) relative to their proportion among all ERE families (71.45%), revealing an increased tissue specificity of LTR sequences compared to LINEs and SINEs (Fig. 2b). Finally, TREs’ expression was typically restricted to fewer tissues than TRGs, with 89.5% of TREs (111/124) being tissue-specific (Fig. 2c, Additional file 1: Table S3). Altogether, these results show that ERE expression in healthy human tissues is widespread but not homogenous. Indeed, 124 ERE families, most of which are LTR elements with low copy numbers, showed tissue-specific expression.

### Impact of the *AIRE* gene on ERE expression in mTECs

**Fig. 3:**
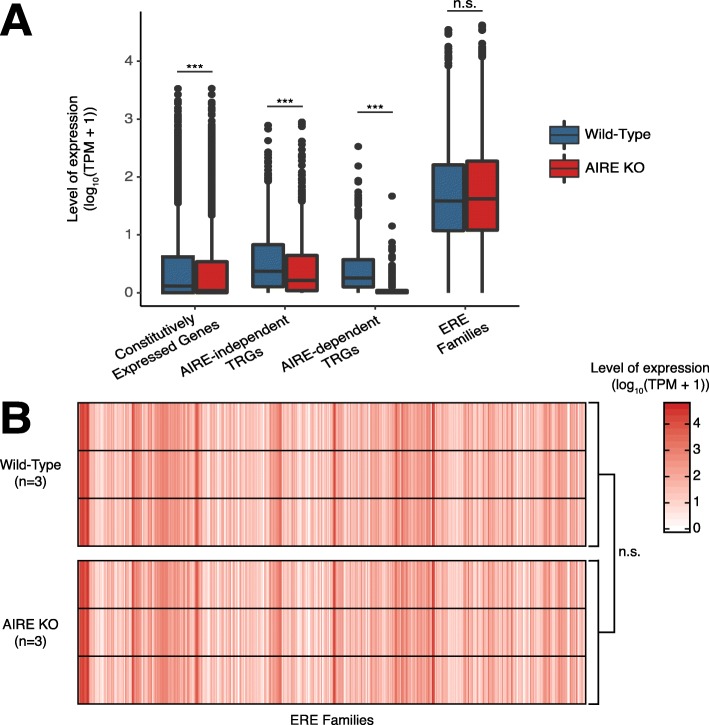
ERE expression is independent of AIRE in mouse mTECs. **a** Boxplot showing the expression levels of constitutively expressed genes, AIRE-dependent TRGs, AIRE-independent TRGs (lists of genes based on St-Pierre et al. [29]), and ERE families in wild-type (*n* = 3) and AIRE knock-out (*n* = 3) mice. **b** Heatmap depicting the expression levels of ERE families in each replicate of wild-type and AIRE knock-out murine mTECs. A Mann-Whitney test was used for statistical analysis in both panels; n.s. not significant (*P* > 0.05, ***P ≤ 0.001)

Out of the three tissues with high ERE expression (Fig. 1), two express no or barely detectable MHC I molecules (testis and ESCs, respectively), whereas mTECs express standard levels of MHC I [55–57]. Promiscuous expression of genomic sequences is a quintessential feature of mTECs that is driven in part by the *AIRE* gene and also by other genes whose identity is still debated [58]. Since the role of mTECs is to induce tolerance to the MAPs that they display, EREs expressed in mTECs could be tolerogenic. However, T cell-mediated responses towards EREs were previously observed, suggesting that the establishment of central tolerance towards EREs in the thymus is incomplete [59, 60]. Therefore, we next investigated the contribution of the AIRE transcription factor to ERE expression in mTECs. To do so, we quantified the expression of ERE families as well as canonical genes in mTECs extracted from wild-type and AIRE knock-out mice previously reported [29]. Canonical genes were sorted in three categories based on St-Pierre et al. [29]: (i) constitutively expressed genes, (ii) AIRE-independent TRGs, and (iii) AIRE-dependent TRGs. As expected, expression of AIRE-dependent TRGs significantly decreased in the absence of AIRE, whereas constitutively expressed genes and AIRE-independent TRGs were minimally affected by AIRE depletion (Fig. 3a). Strikingly, global ERE expression was independent of AIRE since it was unchanged in AIRE knock-out relative to wild-type mice (Fig. 3a). Furthermore, computing Mann-Whitney tests for each ERE family revealed that the absence of AIRE did not affect the expression of any ERE family (Fig. 3b). Hence, the expression of all ERE families was independent of AIRE in mTECs.

### Translation of ERE transcripts

**Fig. 4:**
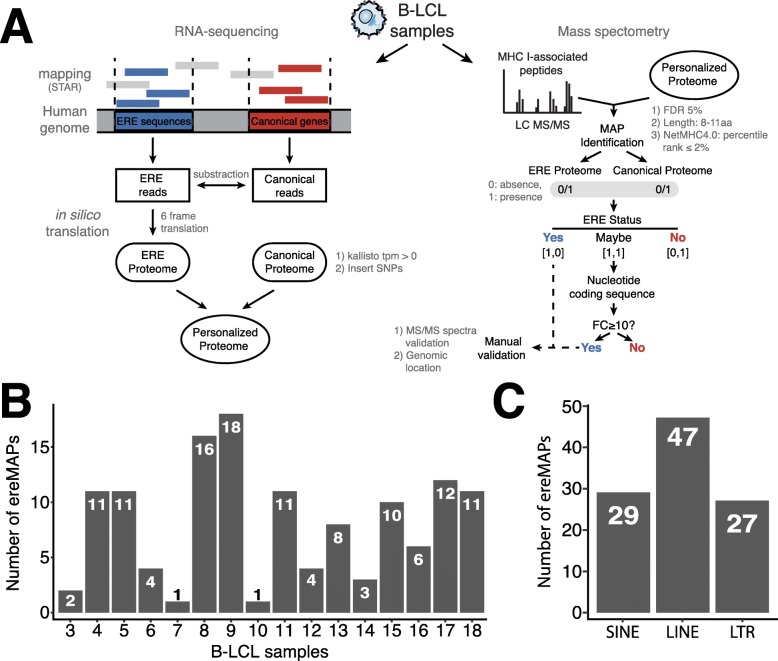
ERE sequences are translated and contribute to the immunopeptidome of B-LCLs. **a** Schematic depicting how the personalized proteome of each B-LCL sample was generated. The personalized proteome was generated by combining the ERE and the canonical proteomes and then used to identify MAPs by MS. MAPs were annotated to keep only ereMAPs. **b**, **c** Barplots showing the number of ereMAPs identified in B-LCL samples separated by **b** individual samples analyzed and **c** according to the three main groups of EREs

We next sought to determine whether some ERE transcripts are translated in healthy cells. However, the identification of EREs by MS can be challenging due to their inherently low abundance in the corresponding proteome and the lack of appropriate protein databases for large-scale searches. We therefore decided to investigate the contribution of EREs to the immunopeptidome, which is mainly composed of peptides derived from rapidly degraded proteins [61, 62]. To do so, we reanalyzed previously reported transcriptomic and immunopeptidomic data from 16 B-lymphoblastoid cell lines (B-LCL) (Additional file 1: Table S4) [34]. As conventional approaches do not include ERE sequences, we developed a proteogenomic workflow combining RNA sequencing and MS to enable ereMAP identification (Fig. 4a, Additional file 2: Fig. S3). Briefly, we generated for each B-LCL a personalized proteome that contained only the sample’s expressed sequences as well as its polymorphisms. Canonical and ERE RNA sequences were translated in silico and concatenated to generate a personalized proteome that was used to identify MAPs in MS analyses (Fig. 4a). For each MAP identified, we retrieved the peptide’s coding sequence and proceeded to its annotation. Two categories of peptides were kept as ereMAP candidates to be further manually validated: (i) peptides that were only seen in the ERE proteome and (ii) peptides seen in both the ERE and canonical proteomes (“maybe” candidates) and for which the occurrence of the coding sequences was at least 10-fold higher in ERE reads compared to canonical reads.

Our proteogenomic approach enabled the identification of 129 ereMAPs in the 16 B-LCL samples analyzed, revealing that ERE sequences are translated in non-neoplastic cells (Fig. 4b). Of those, 103 were non-redundant, confirming that ereMAPs can be shared by multiple individuals (Additional file 1: Table S5). Of course, the extent of interindividual sharing would be considerably greater in cohorts of HLA-matched individuals since various HLA allotypes present different sets of MAPs [61]. Profiling of ereMAPs’ RNA expression in healthy human tissues showed that 26% (27/103) of ereMAPs’ coding sequences were expressed at high levels by multiple tissues (Additional file 2: Fig. S4). Hence, since highly expressed transcripts are preferential sources of MAPs [34], ereMAPs derived from abundant transcripts could be presented on the surface of a wide range of tissues (Additional file 2: Fig. S4). We also observed that ereMAPs were generated by the three main groups of ERE sequences (SINE, LINE, LTR), confirming that they all have the potential to be translated in healthy cells (Fig. 4c). As EREs are frequently dysregulated in cancer cells, we quantified the RNA expression of our ereMAPs (identified in B-LCLs) in 12 cohorts from TCGA (Additional file 2: Fig. S5A). Strikingly, the majority of ereMAPs (94/103, 91.3%) identified in B-LCLs were expressed at similar levels by healthy and cancer cells (Additional file 2: Fig. S5B), and ereMAPs’ RNA expression in cancer cells did not correlate with DNA methylation levels (Additional file 2: Fig. S5C). Additionally, applying our proteogenomic workflow to an ovarian cancer cell line (OVCAR-3) enabled the identification of 5 ereMAPs, including one peptide (TPRHIIVRF) also presented by B-LCL samples (Additional file 1: Table S6). Together, these proteogenomic analyses show that several EREs are translated and generate ereMAPs in B-LCLs, and suggest that this is also the case in a wide range of healthy and neoplastic human tissues.

### High expression of intronic regions is the main source of ereMAPs

**Fig. 5:**
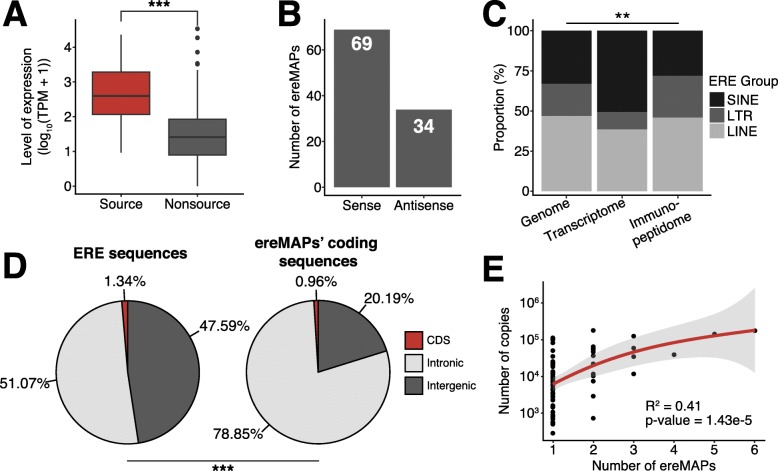
Sense transcription of intronic EREs is the main source of ereMAPs. **a** Boxplot showing the mean expression levels (log_10_(TPM + 1)) of ERE families that are source or non-source of ereMAPs in B-LCLs (Mann-Whitney test, ***P ≤ 0.001). **b** Barplot showing the number of ereMAPs generated by sense or antisense transcription of ERE sequences. **c** Stacked barplot depicting the proportions of LINE, LTR, and SINE groups in the genome, transcriptome, and immunopeptidome. Statistical significance was computed with a chi-squared test (**P ≤ 0.01). **d** Pie charts depicting the percentages of all ERE sequences (left) and of ereMAP-coding sequences (right) that are localized in intergenic regions, introns, or coding sequences (chi-squared test, ***P ≤ 0.001). **e** Scatterplot showing the Kendall tau correlation between the number of ereMAPs generated by each ERE family and the number of copies of the ERE family’s sequence in the human genome based on RepeatMasker annotations

We next investigated the mechanisms leading to the presentation of ereMAPs on the cell surface. First, we noted that ereMAPs preferentially derived from highly expressed ERE transcripts (Fig. 5a). For the majority of ereMAPs, this transcription was in the same sense as the ERE sequence in the genome, but ~ 30% of ereMAPs (34/103) resulted from antisense transcription (Fig. 5b), which is common for EREs [63–65]. Even though ereMAPs were generated by the three main groups of EREs (Fig. 4c), the relative frequency of LTR translation was higher than that of LINEs and SINEs (Fig. 5c). Indeed, the representation of LTRs in the immunopeptidome was superior to the space they occupy in the genome or their abundance in the transcriptome (Fig. 5c). Additionally, intronic EREs were a preferential source of ereMAPs: while 51% of EREs were intronic, ~ 79% of ereMAPs derived from intronic EREs (Fig. 5d). Finally, we noted that some ERE families generated several distinct ereMAPs (Additional file 1: Table S5). This can be explained in part by variations in the genomic space occupied by the various ERE families. Indeed, we observed a moderate, yet significant, correlation between the number of genomic copies and the number of ereMAPs (Fig. 5e). Altogether, these results demonstrate that (i) ereMAPs are generated by both sense and antisense transcripts that are preferentially located in introns and expressed at high levels, and (ii) generation of ereMAPs is enhanced when a family belongs to the LTR group occupying a large genomic space.

### ereMAPs have a viral-like amino acid composition

**Fig. 6:**
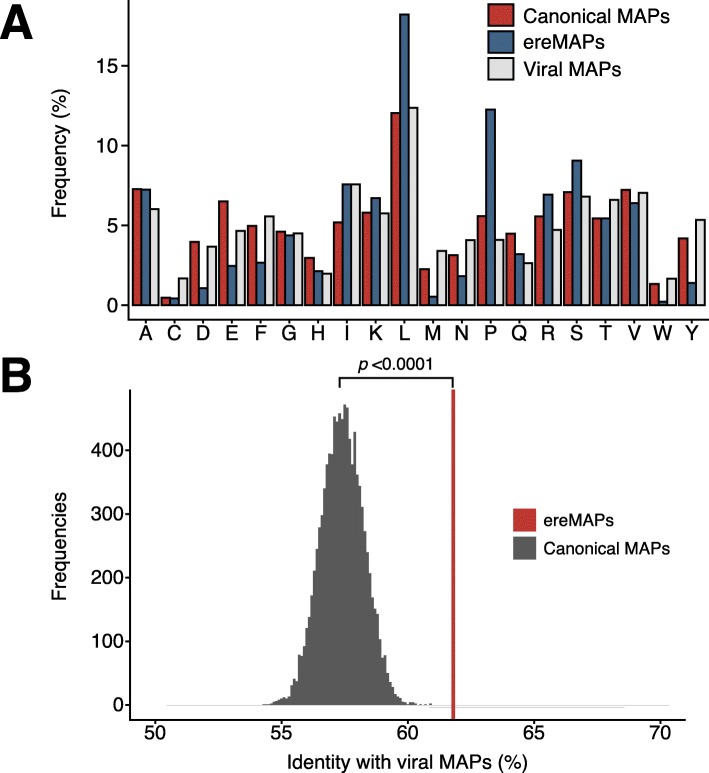
Endogenous retroelements retained sequence homology with viruses. **a** Barplot showing the frequencies of each amino acid in ereMAPs, viral MAPs, and human canonical MAPs. Abbreviations for amino acids: Y, tyrosine; W, tryptophan; V, valine; T, threonine; S, serine; R, arginine; Q, glutamine; P, proline; N, asparagine; M, methionine; L, leucine; K, lysine; I, isoleucine; H, histidine; G, glycine; F, phenylalanine; E, glutamic acid; D, aspartic acid; C, cysteine; A, alanine. **b** Human canonical MAPs and ereMAPs were aligned to a database of viral peptides using BLAST, and the percentage of identity of their sequences with viral peptides was computed. The red line represents the average percentage of identity of ereMAPs with viral MAPs. A bootstrap procedure was used to calculate the percentage of identity of 10,000 sets of 103 randomly selected human canonical MAPs with viral MAPs. *P* value was calculated as the number of times the bootstrap distribution had a higher percentage of identity with viral MAPs than ereMAPs (*P* < 0.0001)

We next asked to what extent ereMAPs and their coding transcripts might retain some traces of their phylogeny (“viral features”). We found conspicuous differences between amino acid frequencies in ereMAPs relative to both viral MAPs and canonical human MAPs listed in the Immune Epitope Database (Fig. 6a). Indeed, ereMAPs showed a lower abundance of multiple amino acids (aspartic and glutamic acids, phenylalanine, methionine, asparagine, and tryptophan) and higher frequencies of leucine (L) and proline (P) residues. ereMAPs had therefore a less balanced (i.e., more skewed) amino acid composition. Furthermore, analysis of amino acid usage at individual MAP positions revealed that, relative to human MAPs, some residues were specifically enriched in ERE and viral MAPs, such as arginine (R) in P5 of 8-amino-acid-long MAPs (Additional file 2: Fig. S6). We therefore aligned ereMAPs sequences to the viral MAP dataset using BLAST and calculated the average percentage of identity between ereMAPs and viral MAPs. We then compared this result with a bootstrap distribution (10,000 iterations) of randomly selected canonical MAPs that were also aligned to the viral MAP dataset (Fig. 6b). This analysis revealed that ereMAPs had a significantly higher percentage of identity with viral MAPs than all 10,000 randomly selected sets of canonical MAPs. Finally, we investigated if the viral features of ereMAPs might confer them the ability to activate CD8 T cells. First, immunogenicity prediction using the Repitope algorithm showed that ereMAPs have significantly higher immunogenicity scores than canonical MAPs presented in the thymus (Additional file 2: Fig. S7A). Additionally, IFNγ ELISpot assays demonstrated that two cancer-specific ereMAPs (i.e., not expressed by mTECs), identified by Laumont et al. [27] on B-ALL samples, have the ability to activate CD8 T cells (Additional file 2: Fig. S7B, C). Hence, ereMAPs clearly retain features that reflect their viral origin, conferring them the ability to elicit CD8 T cell responses when they are not expressed in mTECs.

## Discussion

Hundreds of scientific articles have alluded to the potential implication of EREs in various human diseases, particularly cancer and autoimmunity [2, 66–71]. We therefore felt compelled to draw the global landscape of ERE expression in human somatic cells. One salient point emerging from this atlas is that ERE expression in somatic tissues is more pervasive and heterogeneous than anticipated. All tissues express EREs, but the breadth and magnitude of ERE expression are very heterogeneous from one tissue to another. Thus, we identified 124 ERE families expressed in a tissue-restricted fashion, most of which were LTR elements. LTRs can act as promoters and enhancers to stimulate gene expression [17, 19], and some LTR families are tissue-specifically enriched in intronic enhancer regions containing transcription factor binding sites [72]. Our work therefore suggests that EREs, and more particularly LTRs, may regulate gene expression in a wide range of somatic tissues. In future experiments, single-cell analyses might unveil a further level of heterogeneity that we could not capture by global tissue expression profiling. It was previously reported that EREs were expressed at high levels in two MHC I-deficient cell types: ESCs and testis [73, 74]. That similar levels of expression were found in mTECs for the three major groups of EREs (LINE, SINE, and LTR) (Fig. 1) is remarkable and raises fundamental questions as to the mechanism and role of ERE expression in mTECs. The key role of mTECs is to induce central immune tolerance to a vast repertoire of self-peptides displayed by somatic tissues [58, 75]. Given the large-scale expression of EREs in peripheral tissues highlighted in the present report, we speculate that it may be important for gnathostomes to be tolerant to a wide array of ERE-derived antigens. As a corollary, when EREs are overexpressed, for instance in cancer cells [76, 77], only those that are not expressed in mTECs may be immunogenic. Induction of tolerance to the multitude of self-peptides depends on the unique ability of mTECs to promiscuously express thousands of otherwise tissue-specific genes [78, 79]. Promiscuous gene expression in mTECs is driven in part by *AIRE* and in part by other genes whose identity is unresolved, which may include *FEZF2* as well as genes involved in DNA methylation, histone modification, and RNA splicing [29, 58, 80–82]. Our data clearly show that the overexpression of numerous ERE families in mTECs is entirely AIRE-independent (Fig. 3). This observation underscores the relevance of further studies on the mechanisms of AIRE-independent promiscuous gene expression in mTECs.

A notable finding was that our MS analyses identified ereMAPs derived from LINEs (*n* = 47), SINEs (*n* = 29), and LTRs (*n* = 27). This means that these EREs are translated and produce peptides that are adequately processed for presentation by MHC I molecules. Our analyses suggest that LTRs have a superior ability to generate MAPs. As SINEs do not contain protein-coding sequences, they were expected to generate fewer peptides. However, the reason why LTRs would be more efficiently translated than LINEs remains elusive but might include codon usage and sequence conservation. A few ereMAPs have previously been identified in cancer cells [27, 70, 77]. The presence of ereMAPs on normal cells means that the mere identification of ereMAPs on cancer cells could not be sufficient to infer that these MAPs are cancer-specific nor immunogenic. Nevertheless, we have previously shown in mice that some ereMAPs are truly cancer-specific and immunogenic and can elicit protective anti-tumor responses [27]. Furthermore, compelling evidence has been reported that some LTRs can generate immunogenic ereMAPs in clear cell renal cell carcinoma in humans [67]. These studies coupled to our findings that ereMAPs (i) retain viral-like features (Fig. 6) and (ii) can be recognized by CD8 T cells (Additional file 2: Fig. S7B and C) suggest that ereMAPs may represent particularly attractive targets for the development of cancer vaccines. In line with this, we must also emphasize that the number of translated EREs is certainly superior to the number of ereMAPs identified in our study: (i) collectively, our 16 B-LCLs expressed 39 MHC I allotypes out of the thousands that can be found in human populations (Additional file 1: Table S5), and (ii) like canonical proteins [34], some translated EREs may not generate MAPs.

We anticipate that the biogenesis of ereMAPs in normal and neoplastic cells will be a fertile field of investigation. First, several observations suggest that the landscape of ereMAPs is highly diversified: (i) the MAP repertoire is shaped by several cell type-specific variations in gene expression [83], and (ii) ERE transcription is highly heterogeneous among various cell types (Fig. 1) and can be drastically affected by neoplastic transformation [84]. The processing of ereMAPs is also intriguing. Indeed, following their integration in human genomes, EREs have undergone several rounds of mutation and truncation and very few have previously been shown to be translated [2, 85]. Because ERE sequences are degenerate, they are not expected to yield stable polypeptides. However, MAPs preferentially derive from rapidly degraded unstable peptides, commonly referred to as defective ribosomal products [62]. We therefore hypothesize that for most EREs, translation may yield ereMAPs but not stable long-lived proteins. In other words, the products of ERE translation may be detectable only in the immunopeptidome and not in the proteome.

## Conclusions

In summary, transcriptomic analysis demonstrated that ERE expression is heterogeneous in healthy human tissues, with a higher expression in mTECs, ESCs, and testis than in other tissues. mTECs are the sole normal human cells that express high levels of both EREs and MHC I molecules. In mutant mice, we report that the exceptional expression of EREs in mTECs is AIRE-independent. We also identified ERE families expressed in a tissue-restricted manner, revealing that most healthy human tissues have a unique ERE signature. MS analyses of 16 B-LCL samples enabled the identification of 103 non-redundant ereMAPs, showing that EREs contribute to the immunopeptidome of healthy cells. Interestingly, sharing of ereMAPs by multiple B-LCL samples was observed, and ereMAPs’ coding sequences are expressed at similar levels in other somatic tissues, suggesting that ereMAPs could also be presented by other cell types. Finally, we found that ereMAPs bear strong homology to viral MAPs and therefore have the potential to be particularly immunogenic. We hope that this work will serve as a reference in further studies on EREs in various physiological and pathological conditions.

## Supplementary information

**Additional file 1: Table S1.** Accession numbers of RNA-seq data used in this study. **Table S2.** Expression levels of ERE families in human tissues. **Table S3.** Tissue specificities of ERE families. **Table S4.** Information concerning samples used for immunopeptidomic analyses. **Table S5.** Characteristics of ereMAPs identified in B-LCL samples. **Table S6.** Characteristics of ereMAPs identified in OVCAR-3 cells.

**Additional file 2: Figure S1.** Comparison of ERE expression between mTECs and other cell types. **Figure S2.** Quintile ranking of ERE families in healthy human tissues. **Figure S3.** Manual validation of ereMAPs’ nucleotide coding sequence in the human genome. **Figure S4.** Expression of ereMAPs’ coding sequences in healthy human tissues. **Figure S5**. Expression profiling of B-LCL ereMAPs in cancer. **Figure S6.** Comparison of amino acid usage of ERE-derived, viral and human MAPs. **Figure S7.** Assessment of ERE-derived MAPs’ immunogenicity.

## Data Availability

mTECs’ RNA sequencing datasets generated during this study are available on GEO as GSE127826 (BioProject accession number: PRJNA525591) [28]. Transcriptomic data of four additional mTEC samples, previously reported by Laumont et al. [27], are publicly available on GEO (BioProject accession number: PRJNA525590). ESCs’ transcriptomic data from Lister et al. [24] are available on the short read archive (Accessions: SRR488684 and SRR488685). RNA-seq data of purified hematopoietic cells were obtained from the Gene Expression Omnibus (GEO) (projects PRJNA384650 and PRJNA225999) [25, 26]. RNA sequencing data of WT and AIRE-deficient mice were reported by St-Pierre et al. [29]. Transcriptomic and immunopeptidomic data of B-LCL samples from Pearson et al. [34] were downloaded from GEO (BioProject accession number: PRJNA286122) and the PRIDE Archive (Project PXD004023), respectively. Transcriptomic and immunopeptidomic data of the OVCAR-3 cell line are available on GEO as GSE147570 (BioProject accession number: PRJNA615537) [41] and the PRIDE Archive database (Project PXD018124) [42], respectively.

## Abbreviations

AIRE: Autoimmune regulator
B-LCL: B-lymphoblastoid cell line
DC: Dendritic cell
ELISpot: Enzyme-linked immunospot
ERE: Endogenous retroelements
ereMAP: ERE-derived MAP
ESC: Embryonic stem cells
FDR: False discovery rate
GEO: Gene Expression Omnibus
GTEx: Genotype-Tissue Expression
IFNγ: Interferon-γ
LINE: Long interspersed nuclear element
LTR: Long terminal repeat
MCS: MAP-coding sequence
MAP: MHC I-associated peptide
mTEC: Medullary thymic epithelial cells
MS: Mass spectrometry
SINE: Short interspersed nuclear element
TPM: Transcripts per million
TRE: Tissue-restricted ERE
TRG: Tissue-restricted gene
WT: Wild-type
KZFP: KRAB zinc finger protein
