# Supplementary material for: Widespread and tissue-specific expression of endogenous retroelements in human somatic tissues
Source: Genome Med. 2020 Apr 28;12:40. doi: 10.1186/s13073-020-00740-7 (PMC7189544; doi:10.1186/s13073-020-00740-7)
Supplement: Supplementary file 2 — Additional file 2: Figure S1. Comparison of ERE expression between mTECs and other cell types. Figure S2. Quintile ranking of ERE families in healthy human tissues. Figure S3. Manual validation of ereMAPs’ nucleotide coding sequence in the human genome. Figure S4. Expression of ereMAPs’ coding sequences in healthy human tissues. Figure S5. Expression profiling of B-LCL ereMAPs in cancer. Figure S6. Comparison of amino acid usage of ERE-derived, viral and human MAPs. Figure S7. Assessment of ERE-derived MAPs’ immunogenicity. [file 13073_2020_740_MOESM2_ESM.pdf]

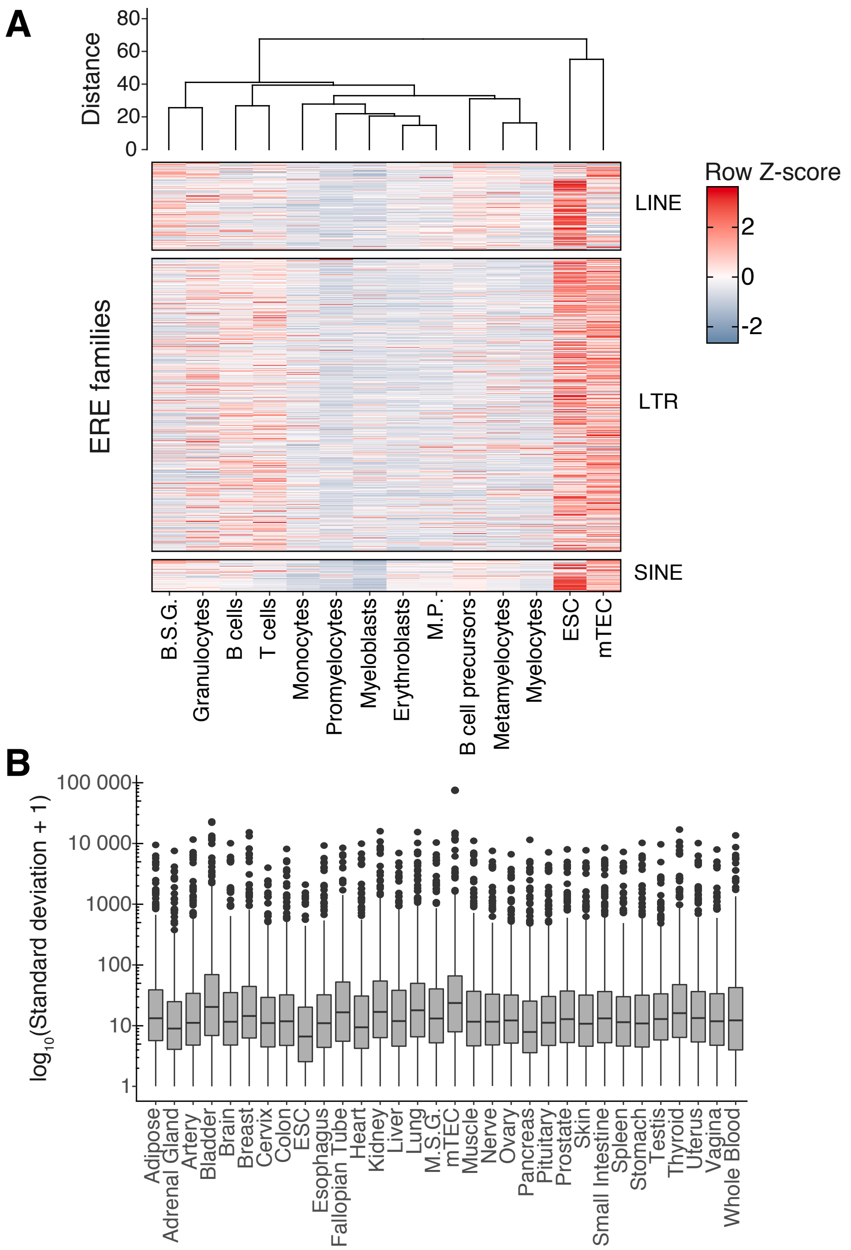

Figure S1. **Comparison of ERE expression between mTECs and other cell types.** (A) Hierarchical clustering of mTECs and multiple hematopoietic cell types based on the expression levels of the 809 ERE families sorted in LINE, LTR and SINE. For each cell type, the mean expression of ERE families was computed among available samples. Row Z-scores were then determined for each ERE family across cell types. (B) Low

interindividual variation in ERE families' expression. Boxplot depicting the log-transformed value of the standard deviation of the expression of each ERE family between samples for the 32 healthy human tissues analyzed. Abbreviations: B.S.G.: Band segmented granulocytes; M.P.: Myelomonocytic progenitors; M.S.G.: Minor salivary gland.

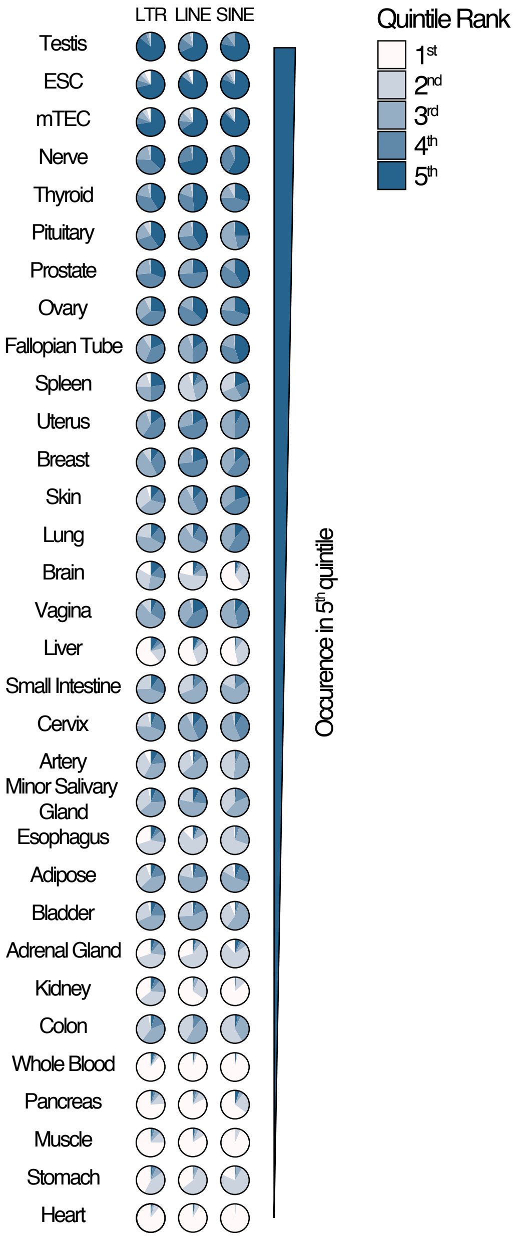

Figure S2. Quintile ranking of ERE families in healthy human tissues. Pie charts represent the percentage of LTR, LINE and SINE families that were assigned to each quintile for the 32 healthy human tissues analyzed. Tissues were sorted based on the number of ERE families that were assigned to the 5th quintile.

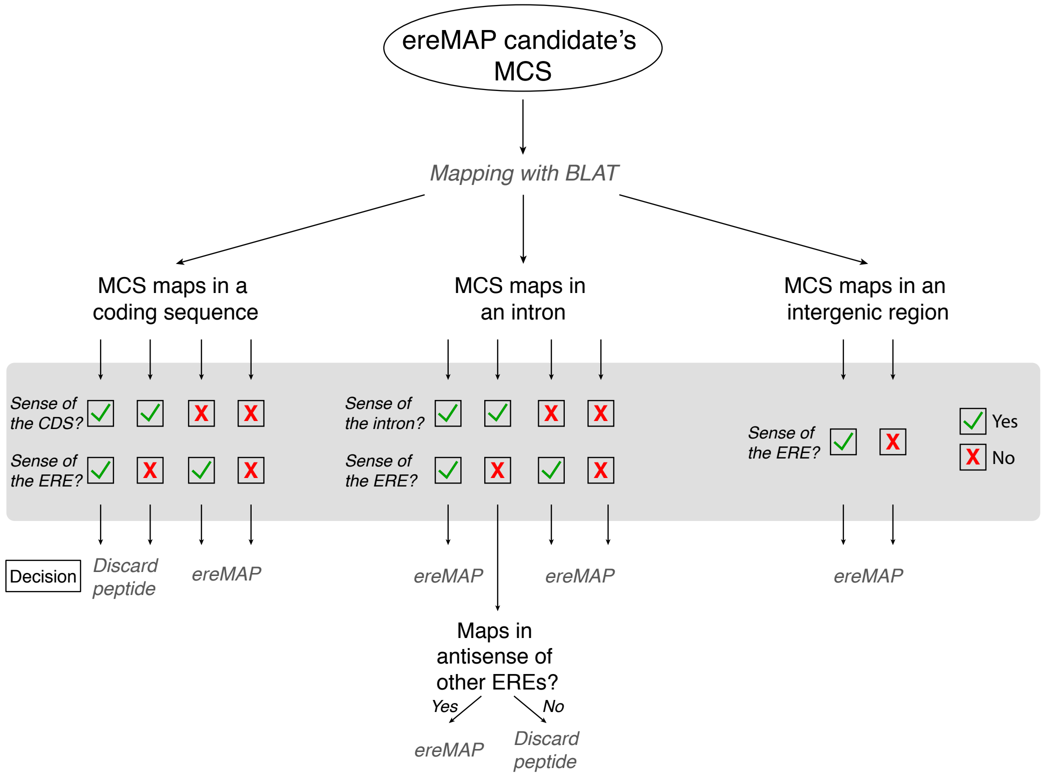

Figure S3. **Manual validation of ereMAPs' nucleotide coding sequence in the human genome**. Flowchart depicting the decision tree for each ereMAP candidate during manual validation in IGV. After mapping of the peptide's coding sequence on the human genome with BLAT, candidates were considered as ereMAPs or discarded based on the orientation of the peptide's coding sequence towards the ERE sequence and other genomic regions (CDS, introns).

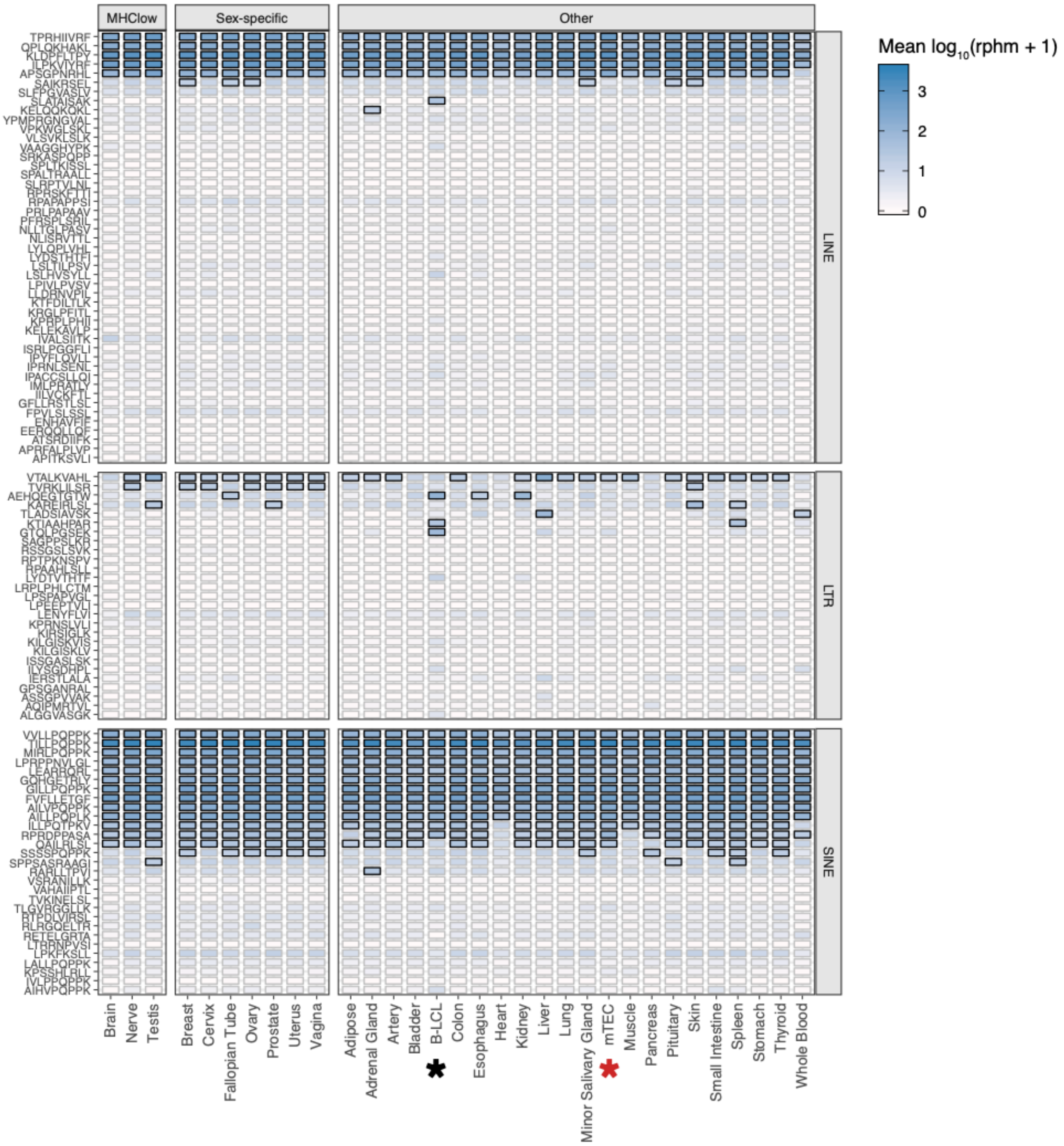

Figure S4. Expression of ereMAPs' coding sequences in healthy human tissues. Heatmap showing the average expression, in reads per hundred million reads sequenced (rphm), of ereMAPs' coding sequences in 32 human healthy tissues/cell types (see Table S1). Peptides were sorted based on the group of the ERE sequence generating the peptide (LINE, LTR or SINE). Positive tissues (rphm > 10) are shown with bold squares. B-LCL and mTECs are indicated with black and red stars, respectively.

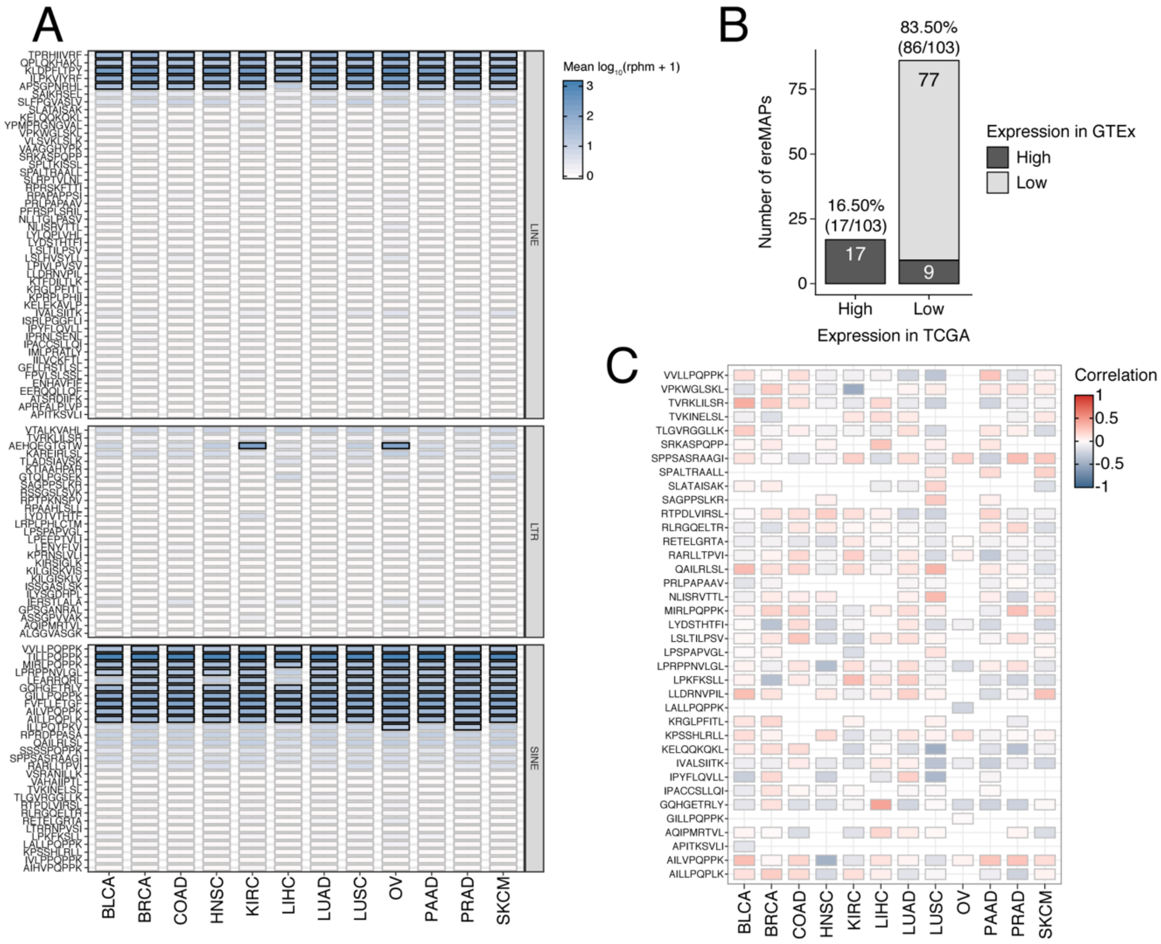

Figure S5. Expression profiling of B-LCL ereMAPs in cancer. (A) Heatmap showing the average expression, in reads per hundred million reads sequenced (rphm), of B-LCL ereMAPs' coding sequences in 12 cohorts of cancers from TCGA. Peptides were sorted based on the group of the ERE sequence generating the peptide (LINE, LTR or SINE). Positive tissues (rphm > 10) are shown with bold squares. (B) Barplot depicting the number of B-LCL ereMAPs with high (above threshold in ≥2 cancer types) or low expression in TCGA cohorts. Shades of grey show the expression of ereMAPs in healthy tissues from GTEx (high if expression is above threshold in ≥2 tissues, otherwise expression is defined as low). (C) Heatmap showing the Pearson correlation between ereMAPs' RNA expression and DNA methylation level. Abbreviations for TCGA cohorts: BLCA, urothelial bladder

carcinoma; BRCA, breast invasive carcinoma; COAD, colon adenocarcinoma; HNSC, head-neck squamous cell carcinoma; KIRC, kidney renal clear cell carcinoma; LIHC, liver hepatocellular carcinoma; LUAD, lung adenocarcinoma; LUSC, lung squamous cell carcinoma; OV, ovarian cancer; PAAD, pancreatic adenocarcinoma; PRAD, prostate adenocarcinoma; SKCM, skin cutaneous melanoma.

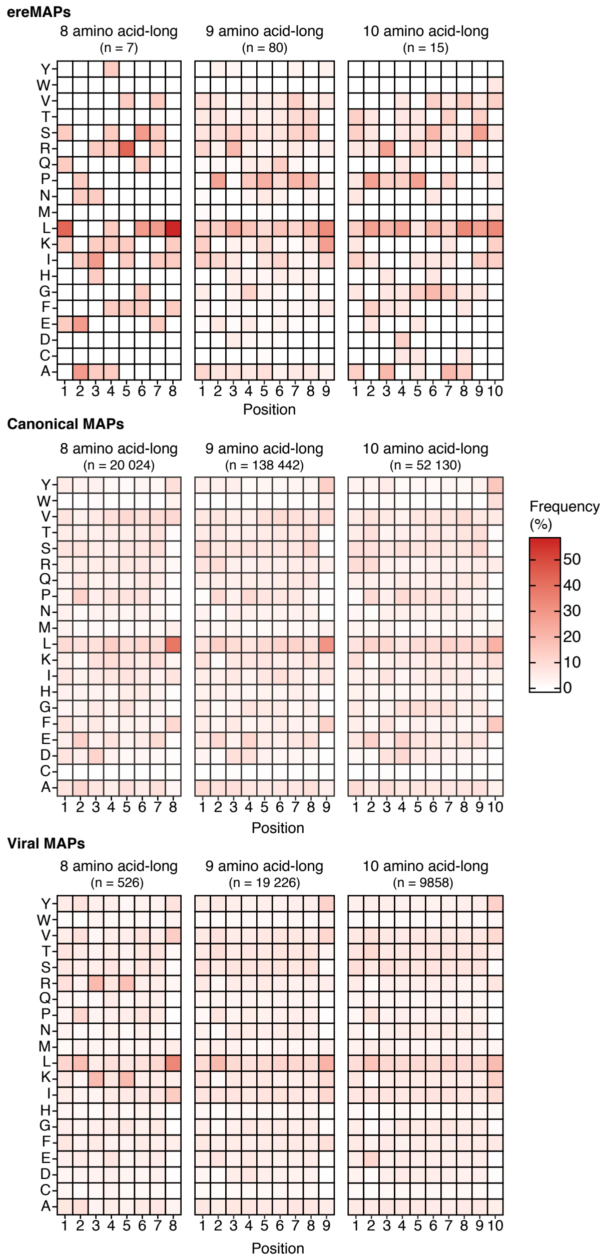

Figure S6. **Comparison of amino acid usage of ERE-derived, viral and human MAPs**. Heatmaps showing amino acid frequencies at all positions of 8, 9 and 10 amino acid-long peptides for ereMAPs (top), canonical human (middle) and viral (bottom) MAPs. Abbreviations for amino acids: Y, Tyrosine; W, Tryptophan; V, Valine; T, Threonine; S, Serine; R, Arginine; Q, Glutamine; P, Proline; N, Asparagine; M, Methionine; L, Leucine;

K, Lysine; I, Isoleucine; H, Histidine; G, Glycine; F, Phenylalanine; E, Glutamic Acid; D, Aspartic Acid; C, Cysteine; A, Alanine.

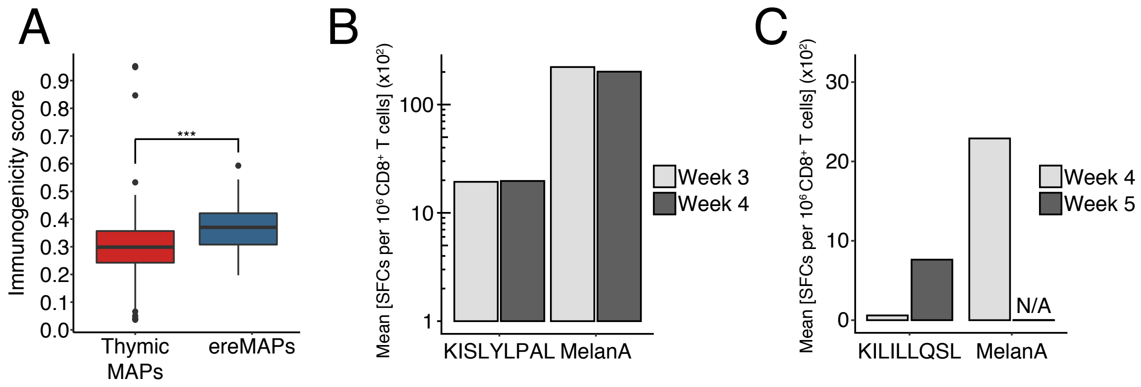

Figure S7. Assessment of ERE-derived MAPs' immunogenicity. (A) Boxplot showing the immunogenicity scores of thymic MAPs and ereMAPs predicted by the Repitope algorithm. Statistical significance was computed with a Mann-Whitney test (****P*≤0.001). (B , C) Barplots showing the numbers of spot-forming cells (SFCs) per 10^6 $CD8^+$ T cells measured by IFNɣ ELISpot assay for two cancer-specific ereMAPs, (B) KISLYLPAL and (C) KILILLQSL, and MelanA as positive control. N/A indicates that the experiment could not be performed due to a limited number of T cells.
